# Food Supplements for Weight Loss: Risk Assessment of Selected Impurities

**DOI:** 10.3390/nu12040954

**Published:** 2020-03-30

**Authors:** Alexandra Figueiredo, Isabel Margarida Costa, Tânia Alexandra Fernandes, Luísa Lima Gonçalves, José Brito

**Affiliations:** 1 Instituto Universitário Egas Moniz (IUEM), Campus Universitário—Quinta da Granja, 2829-511 Monte de Caparica, Portugal; isabelc@egasmoniz.edu.pt (I.M.C.); tania.apf@gmail.com (T.A.F.); lgoncalves@egasmoniz.edu.pt (L.L.G.); jaabrito@netcabo.pt (J.B.); 2 Centro de Investigação Interdisciplinar Egas Moniz (CiiEM), Campus Universitário—Quinta da Granja, 2829-511 Monte de Caparica, Portugal; 3 Instituto de Ciências Biomédicas Abel Salazar (ICBAS), Rua de Jorge Viterbo Ferreira, nº228, 4050-313 Porto, Portugal

**Keywords:** food supplements, trace elements, hazard index

## Abstract

Nowadays, food supplements are widely consumed, often without any medical supervision. In this study, 25 food supplements for weight loss, randomly purchased from five different suppliers in the European Union, were analysed by Wavelength Dispersive X ray Fluorescence spectrometry (WDXRF). The aim of this study was the risk assessment of trace elements and the mixture of elements present in food supplements for weight loss. The obtained Hazard Index (0.11) showed no potential risk of non-carcinogenic effects to human health. However, since humans are frequently exposed by different routes and/or sources to toxic metals, the additional consumption of these products may cause potential toxicological risks that cannot be ignored. In one analysed food supplement (FS), the simultaneous presence of Pb and Mn in high concentrations was detected. In two, FS chromium concentrations were above the reference daily dose. Unconformities were detected between the labelled and the detected values, which emphasises the misinformation of labels. This highlights the need for a deeper surveillance of food supplements.

## 1. Introduction

Vitamins, minerals, nutritional and herbal supplement sales have had a significant increase worldwide, due, in part, to the general misperception that natural indicates harmless [1,2,3]. Greater awareness about healthy ageing and the broader availability of products, as well as trends towards healthy lifestyles and disease prevention lead to an increase in vitamins and food supplements consumption. Besides the growth of self-diagnosis and self-medication by consumers, these products do not need medical prescription and are often consumed without any control or medical supervision, during extended periods of time, which constitutes a concern about the users’ health [4,5,6,7,8].

In the United States (U.S.) it is estimated that approximately 150 million people (one half of the U.S. population) currently use food supplements, with 79% reporting daily consumption and 10% taking five or more different supplements per day [9], with women being the main consumers. In Europe, the use of food supplements is increasing in several countries, however, there is a lack of information with regard to the prevalence and types of food supplements used that can be compared across [10,11].

In Portugal, a study performed in 2006 with the purpose of characterizing the habits of consumption of food supplements, with a sample consisting of 1247 individuals, 60% of whom are habitual consumers and 40% of the individuals are classified as ex-consumers, occasional or non-consumers. The results showed that the use of food supplements mainly occurs among the female population, in younger age groups [12].

Legislation regarding the concentration of elemental impurities in food supplements (FS) is scarce and shows discrepancies between international authorities. Actual requirements for metal impurities in plant-based food supplements imposed by the European Commission (EC), World Health Organization (WHO) and United States Pharmacopeia (USP) only defines limits for As, Cd, Hg and Pb [13,14,15,16,17] (Table 1).

However, the presence of other elements other than the aforementioned may also have adverse effects and potentially put product quality and consumer safety in jeopardy. As USP and European Medicines Agency (EMA) establish limits for several metal impurities in drug products [18,19] (Table 2), it was decided in this study to extend to food supplements the monitoring of Cr, Cu, Ir, Mn, Mo, Ni, Os, Pt, Rh and Ru, all with a validated analytical procedure [20].

At present, the safety of FS and the authenticity of label information is exclusively ensured by the economic operators who place FS on the market. Since these products are usually taken without any medical supervision or counselling and attending to the potential adverse effects of elemental impurities in its composition, it is imperative that these products be placed under the same quality control of pharmaceuticals, regarding FS consumers health [21].

Despite the toxic effects triggered by each element per se, acute exposures to mixtures of elements may induce adverse effects at the same target organ [22,23]. Human health risk assessment related to elemental impurities exposure allows the evaluation of potential adverse effects associated with exposure to these impurities [24]. In practice, risk assessment corresponds to the hazard evaluated from the exposure to a chemical or chemical mixtures [25].

Quantification of risk to human health is performed in terms of non-carcinogenic and carcinogenic risk. In the characterization of non-carcinogenic risk, the United States Environmental Protection Agency (EPA) uses the reference dose factor (RfD) [26], and the values approved by this entity are listed in the Integrated Risk Information System—EPA (IRIS).

Nowadays, the challenge is to develop methodologies to assess the potential risk to health of concerning elemental mixtures, since this is the real scenario of exposure to humans [25]. Therefore, it is crucial to include the study of their adverse effects as an integral part of public health policies [27]. 

Several authors have reported the presence of elemental impurities in several products and the associated hazard risk [28,29,30,31]. 

Nevertheless, to the best of the authors’ knowledge, an assessment of quantitative risk to human health concerning the consumption of food supplements for weight loss has not yet been performed, which demonstrates the innovative nature of the present study.

Besides, FS for weight loss is one of the best-selling dietetic products outside the pharmaceutical distribution network [32,33,34]. 

The aim of this study was the risk assessment of trace elements and the mixture of elements present in food supplements for weight loss.

## 2. Materials and Methods 

### 2.1. Sampling

Twenty five food supplements for weight loss randomly purchased from five different suppliers in Portugal were monitored. All food supplements had a plant-based composition, which may include organic and inorganic compounds. For confidentiality reasons, the studied products were identified with FS nomenclature.

### 2.2. Sample Preparation and Analysis

All samples were solid oral formulations, which were reduced to loose powder and homogenised with glass mortar and pestle. To avoid cross contamination, all glassware and mortars were kept overnight in EDTA (0.1 M) and rinsed several times with Millipore-Q water (18.2 MΩ cm) prior to its use. Samples were preserved in a refrigerator at a temperature of 4 °C, until they were analysed.

Whenever needed, gelatinous coating of the pills was mechanically removed by opening the capsules, and the interior powder content was analysed.

For the preparation of calibration standards, cellulose loose powder was used and at least six concentration levels were considered (in µg/g) in the following ranges: 0–10 (Pb), 0–15 (Ir, Os, Pt, Rh and Ru), 0–30 (Cr, Mo and Ni) and 0–300 (Cu and Mn). The ranges of interest were defined following the recommendations for the studied elements and concentration limits used according to USP and EMA in drug products [18,19] and included the minimum ranges required by ICH guidelines [35]. 

Standards were prepared in triplicate, each of which weighed 5 g consisting of cellulose loose powder spiked with known amounts of each element in study and Millipore-Q water (18.2 MΩ cm). Stock solutions of Cr, Cu, Mn, Ni and Pb, in the concentration range of 0.5–1 mg/mL, were prepared with Millipore-Q water (18.2 MΩ cm), whereas stock solutions at 1 mg/mL of Ir, Mo, Os, Pt, Rh and Ru were purchased from Sigma-Aldrich. After homogenization with magnetic stirring, all standards were dried in an oven at 50 °C over 48 hr and homogenized in a glass mortar and pestle. 

The WDXRF measurements were performed with a 4-kW commercial spectrometer (Bruker S4 Pioneer), using an X ray tube with a rhodium anode and a 75 µm Be end window. For all studied elements, a collimator aperture of 0.46° was used with no filters and a 34 mm diameter collimator mask as well as automatic current reduction allowing for the achievement of the best sensitivity without detector overflow.

High-density polyethylene X ray fluorescence sample cups with a 40-mm diameter assembled with 4 µm prolene film to support the samples were used. The polyethylene cup was placed in steel sample cup holders with an opening diameter of 34 mm. 

### 2.3. Risk Assessment

According to the EPA, if available data is scarce the Hazard Index (HI) to assess cumulative risk is recommended for use for element impurities present in the mixture, assuming the cumulative effect of dose [36]. The calculation of HI does not require the knowledge of action mode of the elements present in the sample and does not consider the interactions between them, it only requires that elements have the same target organ [37,38]. HI determination is based on various elemental constituents of the mixture, for each route of exposure of interest, taking into account a single specific toxic effect or toxicity to a target organ [36]. 

For non-carcinogenic risk the target hazard quocient (THQ) for each element was determined by the ratio between the estimated dose of the element, and the dose level below which there would be no appreciable risk to human health. THQ value is determined by Equation (1) [30].

(1) $$THQ=\frac{EF\times ED\times IR\times C}{RfD\times BW\times AT}$$

where EF is the exposure frequency (days/year); ED is the exposure duration (years), IR is the ingestion rate (kg/day), C is the concentration of the contaminant (mg/kg), RfD is the reference dose (mg/kg/day), BW is body mass (kg), AT is the average time (days). 

The most direct approach to evaluating the potential risk is determination of HI with addition of THQ for each element to be known in the mixture:

(2) $$HI=\overset{i}{\underset{n=1}{\sum }}THQn$$

If HI > 1 there is an unacceptable risk of non-carcinogenic effects on health, while HI < 1 represents an acceptable level of risk [30]. 

The carcinogenic risk estimate is calculated using Equation (3) [39]:

(3) Carcinogenic Risk= Dose×CSF

where CSF corresponds to the cancer slope factor listed in IRIS. EMA considers acceptable or tolerable risk for regulatory purposes the value of 1.0E-6 [40]. However, EPA considers a broader range between 1.0E-6 to 1.0E-4 [41]. The overall risk of effects as a result of exposure to multiple carcinogenic agents is the sum of individual carcinogenic risks of all impurities in the sample.

The values related to the RfD and CSF for the elements under study are shown in Table 3.

## 3. Results and Discussion

The majority of the analysed food supplements presented low levels of the elemental impurities studied as can be observed in Table 4.

The presented values were calculated with concentrations equal or higher than the limit of quantification (LQ) obtained for each element.

In one analysed sample (FS_i_), Mn was detected, although in the label of this supplement there was no mention of manganese in its composition.

The interactions between Mn and Fe play an important role in Mn toxicity. Chronic iron deficiency causes a build-up of higher levels of Mn in the basal ganglia in rats [43] and humans [44]. Since iron deficiency is common in obese and overweight individuals, and the products under analysis are for weight loss, the presence of Mn in these supplements should be considered judiciously [45].

In the same sample, Pb value doubled the limit established by USP (1 µg/g). Pb was also detected in another supplement but within the legal values.

Pb has a high risk of toxicity to humans, with potential effects in the neurological, reproductive, developmental, immune, cardiovascular and renal systems [46]. As reported in literature, fasting and low intake of Fe and Ca promotes greater absorption of Pb [47,48]. Since the analysed products are intended for weight loss, if the target consumers have dietary restrictions, it may increase Pb absorption and turn the consumers of these supplements more vulnerable to lead toxicity. 

The simultaneous presence of high levels of Mn and Pb in the same supplement (FS_i_) increases the concern of the potential toxic effects to human health, since both elements affect the central nervous system.

There is little information about potential adverse effects of co-exposure to Mn and Pb, but existing data suggests that they may have a synergistic neurotoxicity effect. According to Wright and Baccarelli (2007) coadministration of Pb and Mn in animals induces three-fold in Pb levels in the brain when compared with isolated Pb exposure. The joint exposure to lead and manganese resulted in a lower learning ability, relative to that obtained with only Pb exposure. Gestational co-exposure to Pb and Mn not only produces a reduction in fetal weights, but also a decrease in infant brain weight [49].

High levels of Cr were quantified in two samples (FS_ii_ and FS_iii_). However, in these supplements, chromium is labelled as an active ingredient. Therefore, the concentration of this element should be interpreted considering the value of the Cr daily reference dose and not its limit as an impurity.

According to European legislation (Directive 2008/100/EC), chromium may be present in food supplements up to a maximum daily intake of 40 µg/day [50]. On the other hand, the Office of Dietary Supplements from the USA National Institutes of Health [51] establishes different values for Cr intake, according to gender (35 µg/day for adult men and 25 µg/day for adult women), age and pregnancy/lactation. Currently, the European Food Safety Authority (EFSA) Panel on Contaminants in the Food Chain recently derived a Tolerable Daily Intake (TDI) of 300 µg Cr(III)/kg body weight per day from the lowest NOAEL (No Observed Adverse Effect Level) identified in a chronic oral toxicity study in rats [52].The differences between the regulatory authorities in order to a recommended daily dose of Cr are therefore evident.

However, the literature does not unequivocally point toxicity associated with excessive intake of chromium, and does not exclude potential adverse effects with high consumption of this element [53]. In Germany, all the special permissions for the use of chromium picolinate in food supplements were withdrawn in 2001, due to investigations that do not exclude adverse effects on human health [54]. Additionally, the EFSA [55] explicitly excludes chromium picolinate, based on in vitro studies that suggest it may cause DNA damage. However, more recently, EFSA concluded that the use of chromium picolinate was not a concern for the health of its consumers but also noted that recent reviews and evaluations related to Cr (III) show different results in genotoxicity tests. Therefore, EFSA reports that in the future Cr (III) limits should be re-evaluated [56].

In supplement FS_ii_, according to its label, each pill has 34 µg of chromium, which corresponds, according to the package dose instructions, to a daily intake of 136 µg of Cr. This dose was raised to more than three-fold the amount indicated in Directive 2008/100/EC (40 µg/day) and to more than five-fold that recommended by the National Institutes of Health (NIH) value for Cr intake by women (25µg/day), the major weight loss supplement consumers. This was confirmed by the obtained results (28.9 ± 2.0 µg Cr/pill, corresponding to 116.3 ± 3.8 µg Cr/day), as specified in Table 3.

In FS_iii_, chromium was present as chromium picolinate, in a 0.09% concentration, which corresponds to a Cr daily intake of 156 µg, taking into account the labelled instructions. This amount exceeds to almost four-fold the values of Directive 2008/100/EC and to more than six-fold the suggested NIH chromium intake for women. Furthermore, there is an evident discrepancy between labelled values and the obtained results (Table 5). Nevertheless, the detected value (59.9 µg/day) is still above the recommended value.

The detected unconformities found between the measured concentrations of ingredients and the labelled ones were in accordance with several studies, which reported huge differences between the labelled and the measured values in herbal products [57,58]. In 2003, an investigation by the United States Department of Health and Human Services reported that most labels of supplements were misleading and inconsistent and that consumers revealed considerable difficulty understanding the labels correctly. About 85% of inspected labels contained no information about maximum recommended dose [59]. According to several studies, information related to product content in food supplements is frequently inaccurate and incomplete [60,61,62].

For the three aforementioned food supplements, the Hazard Index was determined according to Equation (1), based on 6 months intake, during a period of 2 years. Three distinct values were considered for body mass: 90 kg, 70 kg and 50 kg, where the lower one was based on a conservative approach similar to the calculation recommended by EMA and USP in the determination of elemental impurity limits in drug products.

As can be depicted in Table 6, all HI values were below 1, revealing that there was no potential risk of non-carcinogenic effects on health due to the consumption of the analysed food supplements. However, these results only reflect the intake of Cr, Mn and Pb through food supplements consumption and do not consider the cumulative risk associated with other exposure routes, such as inhalation and environment exposure, neither the eventual risk associated with food, water or other products consumption.

None of the investigated elements were classified by EPA/IRIS as carcinogenic, so there was no CSF established and published, therefore it was not possible to estimate the Carcinogenic risk.

One supplement mentioned chromium as an ingredient in a concentration of 40 µg of Cr per pill. However, its analysis did not reveal the presence of this element, which emphasised the misinformation of the label.

Such as diet or water, these supplements are also potential sources of exposure to toxic elemental impurities. For example, As, Cd, Hg and Pb. may be found in foodstuffs.

Several studies have been published in the literature reporting the presence of elemental impurities in food supplements.

Kauffman et al. (2007) analysed by Inductively coupled plasma mass spectrometry (ICP-MS) 45 samples of pharmaceutical products and FS in order to determine the concentration of Pb in these products. The obtained results revealed that Pb concentration was below the limit imposed by the international bodies for all analysed products [63]. Tumir et al. (2010) analysed by Atomic absorption spectroscopy (AAS) 30 FS, detecting the presence of Cr, Ni and Pb in high concentrations in five of them. In one sample, Cr and Ni were detected in high concentration [64]. In another study performed by Korfali et al. (2013), 33 FS samples of high consumption by the Lebanese population, were analysed using three different techniques (Energy Dispersive X-ray Fluorescence spectrometry (EDXRF), AAS and ICP-MS) in order to determine the presence of As, Cd, Cr, Cu, Fe, Hg, Mo, Mn, Pb, Se and Zn in these products. Approximately 30% of the analysed samples showed Cd concentrations above the established levels and in 62% of the analysed samples the concentration of As were above the established limit [4]. In further research developed by Filipiak-Szok et al. (2014) with 19 samples of food supplements (with asiatic and european plants) analysed by ICP-MS, high levels of Pb was detected in one product and As in another [65]. Dolan et al. (2003) analysed the As, Cd, Hg and Pb contents of 95 FS products using microwave digestion and high-resolution ICP-MS. In 11 of the products, levels of lead found result in exposures that exceed the tolerable intakes of some sensitive segments of the population such as children and women of child-bearing age, particularly pregnant women [66].

Through the research in RASFF it was possible to identify 16 alert notifications, between 2016 and 2020, linked to the high levels of elemental impurities in FS composition. Eight of these are related to the presence of Pb, two alerts report the presence of Zn, and three of them alert to the simultaneous presence of Pb + Al, Pb + Hg, and Pb + Hg + Al. High concentrations of Hg, Ni and As were also detected in three FS. However, it was not possible to know whether these FS correspond to FS for weight loss, since the information given in RASFF is “dietetic foods, food supplements and fortified foods” [67].

## 4. Conclusions

In one analysed food supplement (FS_i_), the simultaneous presence of Pb and Mn in high concentrations was detected. In two FS, chromium concentrations were above the reference daily dose. This study also revealed discrepancies between label doses and the detected values, which discloses the deficiency in quality assessment and the unreliability of the labelling of these products. Therefore, it seems important to claim a more exigent regulation of food supplement labelling and to set the same quality standards for food supplements as for pharmaceuticals, extending concentration limits to other elements than those already imposed, due to the pernicious effects they may have on consumer’s health.

## Figures and Tables

**Table 1:** Current European Commission (EC), World Health Organization (WHO) and United States Pharmacopeia (USP) limits for metals impurities in medicinal products, in μg/g (oral route) [13,14,15,16,17].

Element	EC	WHO	USP
**As**	*	*	1.5
**Cd**	1.0	0.3	0.5
**Hg**	0.1	*	1.5
**Pb**	3.0	10	1.0

* No value assigned.

**Table 2:** Current European Medicines Agency (EMA) and USP limits for metals impurities in pharmaceuticals (oral route) [18,19].

Element	Concentration (μg/g)	PDE (μg/day)
*Cd, Pb*	0.5	5
*As*	1.5	15
*Hg*	3.0	30
*Co*	5.0	50
*Au, Ir, Os, Pd, Pt, Rh, Ru and V*	10	100
*Tl*	0.8	8
*Se, Ag*	15	150
*Ni*	20	200
*Li*	55	550
*Sb*	120	1200
*Ba*	140	1400
*Cu, Mo*	300	3000
*Sn*	600	6000
*Cr*	1100	11,000

PDE—Permitted Daily Exposure.

**Table 3:** Oral Reference dose values (RfD) and Cancer Slope Factor (CSF) listed in IRIS for the elemental impurities under study.

Metals	Oral RfD (mg/kg/day)	CSF (mg/kg/day)^−1^
Cr	1.5	n.d.
Cu	0.04	n.d.
Ir	n.d.	n.d.
Mn	0.14	n.d.
Mo	0.005	n.d.
Ni	0.02	n.d.
Os	n.d.	n.d.
Pb	0.0036 ^a^	n.d.
Pt	n.d.	n.d.
Rh	n.d.	n.d.
Ru	n.d	n.d.

^a^ WHO value [42]; n.d. not determined.

**Table 4:** Metal impurities levels measured in samples of 25 food supplements.

Element	<LQ	Mean ± SD ^a^ (µg/g)	Element Level ^b^ (µg/g)	Established Limit (µg/g)
Cr	22	42.22 ± 16.87	42.54 (22.19; 63.12)	1100
Mn	20	248.08 ± 333.17	99.77 (66.63; 896.08)	n.e.
Mo	17	7.69 ± 1.70	7.13 (5.57; 10.44)	300
Os	22	2.84 ± 0.41	2.84 (2.19; 3.39)	10
Pb	24	2.09 ± 0.37	2.04 (1.74; 2.48)	1 ^c^
Ru	12	14.44 ± 2.75	14.33 (10.06; 19.11)	10

^a^ SD—Standard Deviation; ^b^ Measured element levels, expressed as medians, with minimum and maximum values given in brackets; ^c^ Value establish by USP for Food Supplements; n.e.–not established.

**Table 5:** Comparison between labelled and obtained results for supplements FS_ii_ and FS_iii._

Sample	Label Information			Obtained Results	
	Cr (µg/pill)	Recommended Number Pills/day	Ingestion Rate (µg Cr/day)	Cr (µg/pill)	Ingestion Rate (µg Cr/day)
FS_ii_	34	4	136	28.9 ± 2.0	116.3 ± 3.8
FS_iii_	78	2	156	29.9 ± 0.4	59.9 ± 0.6

**Table 6:** THQ and HI values for FS_i_, FS_iI_ and FS_ii_ (mean ± SD).

Supplement FS_i_				Supplement FS_ii_	Supplement FS_iii_
Body Mass (kg)	THQ (Mn)	THQ (Pb)	HI	HI (Cr)	HI (Cr)
**50**	0.1714 ± 0.0008	0.0156 ± 0.0028	0.1870 ± 0.0035	0.0008 ± 2.5 × 10^−5^	0.0004 ± 3.7 × 10^−6^
**70**	0.1225 ± 0.0006	0.0111 ± 0.0002	0.1336 ± 0.0025	0.0005 ± 1.8 × 10^−5^	0.0003 ± 2.7 × 10^−5^
**90**	0.0952 ± 0.0005	0.0087 ± 0.0015	0.1039 ± 0.0019	0.0004 ± 1.4 × 10^−5^	0.0003 ± 2.1 × 10^−5^
